# Poisoning by Rhus-Toxicodendron

**Published:** 1891-11

**Authors:** W. A. Yingling

**Affiliations:** Nonchalanta, Kansas


### POISONING BY RHUS-TOXICODENDRON.

W. A. YINGLING, M. D., NONCHALANTA, KANSAS.

Apropos the discussion of ivy poisoning in the August number of THE HOMŒOPATHIC PHYSICIAN, page 334, I report the following case:

Miss N. W., æt. twenty-five, single, has been excessively sensitive to ivy poisoning; she could not go within from ten to twenty feet of it, when the dew was on, without being covered with the rash peculiar to that poison vine. She was so much and so often affected that, by the frequent use of sugar of lead, she has a full beard, the envy of many a young man. She was troubled with it in the worst form I ever saw. About three years ago I gave her *Rhus-tox.*$^{3x}$, but used no external applications, which soon relieved her, and from that time she was not so sensitive and could be near it without being poisoned. This spring she was again unfortunate enough to be poisoned, when I gave her *Rhus-tox.*$^{200}$, no external applications, with a positive cure. She can now "handle it, and even crush it in her hands," as she tells me to-day, without any result at all. She had a proving of the remedy soon after taking it, a severe headache with a sensation as though her head would fly off, or go on forward, whenever she stood still, but not so when she would move about or walk.

She is greatly rejoiced and would be still more so if her beard could be removed. Does any one know of a remedy to remove this beard without injury to her?

I have noticed the following remedies vouched for as "almost" or real specifics for the cure of ivy poisoning: Agar., Anacard., Apis, Arn., Bry., Croton-tig., Euphorb., Graph., Indm., Ledum, Nymphea-od., Nymphea-lut., Puls., Rhus-tox., Rhus-ven., Sang., Sep., Spts-nit., Sulph., Verbena-hast.

This contrariety of opinion does not question the homœopathic law of cure, but corroborates the necessity and importance of the selection of the true simillimum in the cure of any complaint. Every remedy is a true *specific* when it is the simillimum. I have cured cases with Rhus-tox. and Puls.

